# Recent Strategies for Ni_3_S_2_-Based Electrocatalysts with Enhanced Hydrogen Evolution Performance: A Tutorial Review

**DOI:** 10.3390/ijms26083771

**Published:** 2025-04-16

**Authors:** Yucheng Shen, Jixing Bai, Huijie Wei, Jun Gu, Qi Cao

**Affiliations:** Key Laboratory of Energy Thermal Conversion and Control of Ministry of Education, School of Energy and Environment, Southeast University, Nanjing 210096, China

**Keywords:** nickel sulfides, hydrogen evolution reaction, structural design, facet engineering, defect engineering

## Abstract

Water electrolysis represents one of the most environmentally friendly methods for hydrogen production, while its overall efficiency is primarily governed by the electrocatalyst. Nickel sulfides, e.g., Ni_3_S_2_, are considered to be highly promising catalysts for the hydrogen evolution reaction (HER) due to their distinctive chemical structure. However, the practical application of Ni_3_S_2_-based electrocatalysts is hindered by unsatisfactory high overpotential in the HER and weakened catalytic performance under alkaline conditions. Therefore, in this regard, further research on Ni_3_S_2_-based catalysts is being carried out to tackle these challenges. This review provides a comprehensive survey of the latest advancements in Ni_3_S_2_-based in improving the HER performance of Ni_3_S_2_-based electrocatalysts. The review may offer some inspiration for the rational design and synthesis of novel transition metal-based catalysts with enhanced water electrolysis performance.

## 1. Introduction

With societal advancement, energy demand continues to rise. As a non-renewable resource, traditional fossil fuels face eventual depletion due to limited reserves. Furthermore, the combustion of fossil fuels generates CO_x_ and SO_x_ emissions, contributing to environmental problems, such as global warming and acid rain [1,2,3,4]. Energy transformation has emerged as a vital trial for the next 30 years, driven by the pressing need to reduce global greenhouse gas emissions. Meanwhile, the development of low-cost renewable energy is crucial for environmental protection and energy security. Hydrogen energy, one of the environmentally friendly and efficient clean energies, can effectively achieve low carbon emissions and substitute fossil energy sources [5]. The calorific value of hydrogen (H_2_) under standard conditions is as high as 1.43 × 10^8^ J/kg, with water as the only combustion product. The product, in turn, could be recycled as the raw material for hydrogen production, with high energy supply and utilization efficiency. Therefore, hydrogen energy development and utilization are key directions in the global energy transition [6,7].

In fact, H_2_ can be divided into green hydrogen, blue hydrogen, and gray hydrogen, according to the amount of carbon emissions generated during the production process and the energy sources used. Gray hydrogen refers to H_2_ produced by the reforming or gasification of fossil fuels, e.g., the steam methane reforming method. Although it has been widely adopted in industrial production because of its low technological barrier and low production cost, this inevitably leads to environmental pollution. Thus, exploring a better hydrogen production method is essential. Blue hydrogen is produced by fossil energy, such as coal, oil, and natural gas, using carbon reduction as the main production method. Amid its production, by-products, such as CO_2_, could be captured by carbon capture technology, achieving low carbon emissions. Green hydrogen refers to H_2_ produced without any carbon emissions through electrolysis of water using electricity generated from renewable energy [8,9,10]. Integrating renewable energy with water electrolysis is highly advantageous, as surplus electricity can be chemically stored as hydrogen, addressing the gap between energy production and demand.

Compared with other technologies, electrolytic water splitting has the advantages of simple equipment, less pollution, high hydrogen purity, and less impurities, and thereby has received extensive research and attention [11,12,13,14,15,16]. However, there is an inevitable overpotential due to the energy barrier, resulting in high power consumption and high production costs. It is known that the overpotential can be reduced by electrocatalysts, thus realizing the purpose of efficient hydrogen production. To address this issue, it is necessary to pay attention to exploring highly reactive electrocatalysts with outstanding durability. Studies have shown that the HER under alkaline or neutral conditions requires the additional cleavage of water molecules, generally requiring higher energy, compared with acidic conditions. In order to improve the catalytic activity under these conditions, it becomes crucial to design precise regulatory strategies from the perspective of reaction kinetics and lowering energy barriers.

Recent studies have shown that noble metal catalysts exhibit good HER, but noble metals are expensive and not suitable for industrial production [17]. Transition metal compounds, with abundant reserves, low cost, and inherent HER activity, have attracted extensive attention as promising alternatives to noble-metal-based catalysts. Among various transition metals, Ni exhibits relatively high catalytic activity and possesses chemical properties similar to those of Pt, making its compounds highly valuable in electrocatalytic HER applications. As a representative nickel-based compound, Ni_3_S_2_ has garnered significant interest due to its unique structural advantages. On the one hand, the high electronegativity of S atoms can modulate the electronic environment around Ni atoms. On the other hand, Ni_3_S_2_ exhibits a band-gap-free electronic structure, along with excellent structural stability and electrical conductivity, positioning it as one of the most promising HER catalysts. However, the strong chemisorption of H* intermediates on Ni_3_S_2_ leads to a relatively high overpotential, hindering its practical applications [18]. Therefore, current research efforts are increasingly focused on further enhancing and optimizing the HER activity of Ni_3_S_2_. Therefore, in this review, we summarize the research progress and modulation strategies of Ni_3_S_2_ and various nickel-based catalysts. It also highlights recent advances in reducing the overpotential of Ni_3_S_2_ by enhancing its intrinsic catalytic activity, increasing active site density, and constructing heterostructures. We hope that this review will offer insights into the synthesis principles of Ni_3_S_2_ and electrocatalyst design, potentially advancing their broader future application.

## 2. Mechanism of HER and Performance Evaluation Parameters

**Figure 1:**
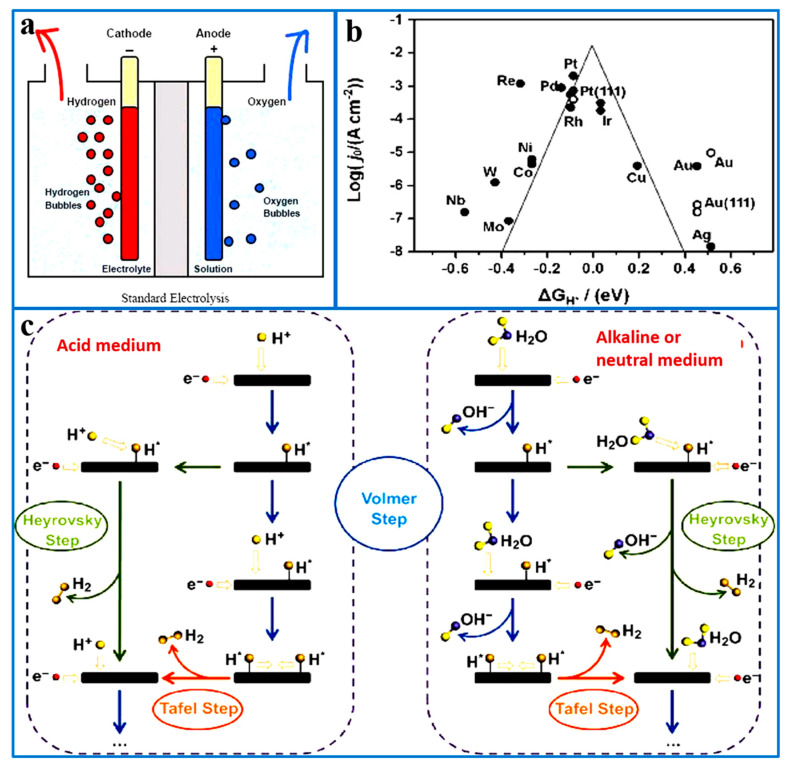
(**a**) Schematic diagram of the water electrolysis device [26]. (**b**) Volcano plots of the relationship between Gibbs free energy and the exchange current density for hydrogen atoms adsorbed by different catalysts. Reproduced with permission [27]. Copyright 2005, Electrochemical Society. (**c**) Schematic diagram of the HER process in an acidic medium, alkaline medium, and neutral medium, respectively. Reproduced with permission [28]. Copyright 2018, Springer Nature.

The electrolytic water splitting reaction involves the HER at the cathode and the OER at the anode, producing H_2_ and O_2_, respectively, as illustrated in Figure 1a [19,20]. In the HER occurring at the cathode, H^+^ is reduced on the cathode surface by accepting electrons, resulting in the formation of H_2_. This process is primarily divided into two steps: first, H^+^ or H_2_O are adsorbed onto the electrode surface through the Volmer reaction; then, the adsorbed H* is released in the form of H_2_ through the desorption process. The desorption process can occur via the Heyrovsky or Tafel reaction, which correspond to two mechanisms of the HER: the Volmer–Heyrovsky mechanism and the Volmer–Tafel mechanism, respectively. Moreover, the HER pathway is influenced by factors such as the pH of the electrolyte, ion types, concentrations, and the catalytic environment. As shown in Figure 1c, these steps exhibit different behaviors under varying pH conditions. Evidently, the HER requires an intermediate hydrogen adsorption step.

Therefore, the catalytic activity of metal catalysts in the HER is typically described by the Gibbs free energy of hydrogen adsorption (∆G_H*_) [18]. A high ∆G_H*_ value (∆G_H*_ > 0) results in reduced hydrogen evolution activity by hindering the H* adsorption on the surface of the catalyst, thereby suppressing the occurrence of the Volmer step. However, a low G_H*_ value (∆G_H*_ < 0) suggests too strong an adsorption capacity of the catalyst for H*, which makes it difficult to desorb H* from the catalyst surface and generate H_2_ [21,22]. According to the Sabatier principle, the numerical difference between the value of ∆G_H*_ and 0 represents the adsorption and desorption capacity of the catalyst surface for H*, and the smaller the difference, the more favorable the HER becomes [23,24]. The ∆G_H*_ of various catalyst materials, calculated using density functional theory (DFT), and the experimentally measured exchange current density are plotted on a coordinate diagram, revealing the volcano relationship between the catalyst’s hydrogen adsorption free energy and exchange current density (Figure 1b). The noble metal-based catalysts are located at the peak of the volcano curve, indicating excellent HER activity [25].

The catalytic performance of electrochemical catalysts is affected by various parameters, such as overpotential (*η*), Tafel slope and exchange current density (*j*_0_), electrochemical activity specific surface area (ECSA), electrochemical impedance spectroscopy (EIS), stability, and others. These parameters could provide important information on kinetics and thermodynamics, serving as the basis for improving the performance of electrocatalysts in a targeted manner. This section briefly introduces the aforementioned parameters.

### 2.1. Overpotential

In electrochemistry, *η* refers to the deviation between the theoretically calculated reduction potential of a half-reaction and the actual potential measured during the redox process. The potential value when the current density reaches 10 mA·cm^−2^ is regarded as the *η* value, evaluating the HER performance [29,30]. Generally, elevated *η* would cause greater electrical energy consumption in the reaction process, negatively affecting the corresponding energy conversion efficiency. Generally, a lower overpotential enhances the hydrogen evolution activity of the catalyst in the HER.

### 2.2. Tafel Slope and Exchange Current Density

The Tafel slope reflects the kinetic parameters during the electrocatalytic reaction. According to the Tafel formula, i.e., *η* = b log*j* + a, the fitted curve describes the relationship between *η* and the current density (*j*) [5,30]. A low Tafel slope value requires a low *η* to increase the same *j*, indicating a high hydrogen evolution activity of the catalyst [31]. The *j*_0_ refers to the microscopic forward or reverse reaction rate of charge transfer on the electrode/electrolyte heterointerface per unit area under the condition of dynamic equilibrium during the HER [32]. In addition, the value of *j*_0_ can be estimated from the value at the intersection of the linear part of the Tafel curve and the *X*-axis [33]. In general, the *j*_0_ is found to be positively correlated with the catalytic efficiency [34,35].

### 2.3. Electrochemical Active Specific Surface Area

The electrochemically active surface area of a catalyst indicates its intrinsic surface exposed to the electrolyte, reflecting the number of active sites in the electrocatalytic reaction. The ECSA value is typically evaluated using the electrochemical double-layer capacitance (C_dl_) test. A higher C_dl_ value generally correlates with a higher ECSA, suggesting better catalytic activity [36].

### 2.4. EIS

EIS is a widely used technique to evaluate the interfacial charge transfer characteristics of electrocatalysts. In HER studies, a lower charge transfer resistance (R_ct_), as indicated by a smaller semicircle in the Nyquist plot, reflects faster electron transfer kinetics and improved catalytic efficiency.

### 2.5. Stability

In addition to activity, stability is a key parameter in assessing performance. It can be quantified by monitoring changes in the current density (or electrode potential) over an extended period (greater than 12 h) under constant electrode potential (or current) conditions. Smaller changes indicate better catalyst stability.

### 2.6. Faradaic Efficiency

The Faradaic efficiency (FE) quantifies the proportion of electrons that contribute to the desired electrochemical reaction within a given system. For the HER, FE is defined as the ratio between the experimentally measured amount of hydrogen and the theoretically calculated hydrogen yield. The theoretical yield is estimated based on the total charge passed, assuming 100% electron-to-product conversion [30,31]. In contrast, the actual hydrogen production is typically determined using either the water displacement method or gas chromatography (GC) analysis.

### 2.7. Mass Activity

Mass activity is an essential performance metric that reflects the catalytic current normalized to the mass of the active material. In the HER, mass activity is typically expressed in units of mA·mg^−1^ and provides insight into how effectively the catalyst can drive the reaction per unit mass. A high mass activity indicates that a smaller quantity of catalyst can achieve comparable or superior electrocatalytic performance, which is critical for cost-effective and scalable hydrogen production.

### 2.8. Specific Activity

Specific activity refers to the current density normalized to the ECSA and is commonly used to evaluate the efficiency of individual active sites. It eliminates the influence of factors such as catalyst loading, surface roughness, and morphological variations, thereby providing a more intrinsic measure of catalytic performance.

### 2.9. Turnover Frequency

The turnover frequency (TOF) can be defined as the number of reactant molecules converted to a specific product per active site per unit time. It reflects the intrinsic catalytic activity of each individual active site and is typically expressed in units of s^−1^.

## 3. Research Status of Ni-Based HER Electrocatalysts

Noble metal-based electrocatalysts face substantial limitations in practical applications, primarily due to the high cost and scarcity of noble metal resources. Therefore, there is a need to develop non-noble metal catalysts that offer excellent HER performance, abundant reserves, and low cost. Non-noble metals are generally categorized into non-metallic and transition metal catalysts. Transition metal catalysts generally exhibit superior electrocatalytic activity compared to non-metal catalysts while being inexpensive and abundant. Therefore, the transition metal catalyst is an excellent electrocatalyst that can be put into industrial production. In terms of the choice of transition metal compounds, Ni-based compounds are considered effective substitutes for noble catalysts. The reasons can be summarized as follows: Ni and Pt belong to the same group in the periodic table, sharing similar chemical properties, and compared with Pt, Ni has low cost and sufficient reserves, so Ni compounds have important research value in the HER [37]. This section mainly introduces the research on the hydrogen evolution performance of nickel-based phosphides, nickel-based oxides, nickel-based hydroxides, and nickel-based sulfides in recent years and compares the advantages and disadvantages of these nickel-based compounds (Table 1).

### 3.1. Nickel-Based Oxides and Hydroxides

As a catalyst for HER, nickel-based oxides have the characteristics of low toxicity, abundant reserves, and low cost. However, the catalytic hydrogen evolution activity of nickel-based oxides is relatively poor because of the poor electrical conductivity of the oxides. Moreover, the crystal electronic structure of nickel-based oxides has too strong adsorption energy for H*, resulting in difficulties in desorbing H*, thereby impairing the generation of H_2_ [37]. Researchers have improved the hydrogen evolution activity of nickel-based oxides by reducing the O content or creating O vacancies. For example, using the calcination reduction method, Feng et al. [43] synthesized NiCo_2_O_4_ catalysts with O vacancies, achieving an overpotential and Tafel slope of only 135 mV and 52 mV dec^−1^, respectively. This is due to the fact that the O vacancies can shift the electronic state density of the Co atom d orbitals, which reduces the binding energy of the chemical bond with the HER intermediate. This reduces the adsorption energy of the active site of the catalyst to the H_2_O barriers to improve HER performance.

Although the hydrogen evolution catalytic activity of nickel-based hydroxides is not optimal, it is easy to adsorb OH* groups in the HER due to the unique electron distribution, thereby promoting water splitting in the Volmer step and the formation of H*, which is used to combine the catalyst with transition metal hydroxides to design more efficient and inexpensive catalysts [33,44]. For example, Yang et al. [45] synthesized MoS_2_/NiCo-LDH materials with high-efficiency hydrogen evolution performance, achieving an overpotential of merely 78 mV and a Tafel slope of 76.6 mV·dec^−1^ under 1M KOH. The synergistic effect between NiCo-LDH and MoS_2_ optimized the chemical adsorption of the intermediate medium (H* and OH*) and promoted the efficiency of water splitting. At the same time, DFT results showed that the Gibbs free energy of MoS_2_/NiCo-LDH for the HER intermediate medium was close to 0, reaching higher hydrogen evolution activity.

### 3.2. Nickel-Based Phosphide

Nickel-based phosphides have good electrical conductivity, mechanical strength, and chemical stability. Its higher HER activity can be attributed to P doping because the high electronegativity of P atoms could attract more electrons around the metal atoms and form the electron configuration. Liu et al. [46] synthesized monodisperse nickel phosphide nanocrystals (Ni_2_P, Ni_5_P_4_, and Ni_12_P_5_) with different atomic ratios of Ni and P, indicating that the hydrogen evolution activity is directly related to the atomic percentage of P. Among them, Ni_5_P_4_, possessing the highest atomic percentage of P (44%), showed excellent hydrogen evolution activity and stability. However, excessive P atoms would prevent the electron movement of metal atoms. Thus, the balance of P atom content is very important. At a moderate atomic ratio of metal atoms to P atoms, transition metal phosphides exhibit high metal-like conductivity [47].

Additionally, the intrinsic catalytic activity of metal phosphides can be enhanced by heteroatom doping. Yang et al. [48] incorporated Mo atoms into the Ni_2_P lattice using nickel foam (NF) as the substrate, and the synthesized Mo-Ni_2_P/NF provided a high HER activity of 10 mA·cm^−2^ at a low overpotential of 82 mV due to the synergistic effect between Mo and Ni atoms. Specifically, Mo doping can markedly improve the catalytic performance of Ni_2_P by improving conductivity, accelerating electron transfer, and creating efficient HER catalytic active sites. Recent research on the electrocatalytic activity of nickel-based phosphides has made great progress and breakthroughs. However, nickel-based phosphides are easily oxidized, resulting in poor stability [49,50]. Therefore, it is still challenging to achieve the goal of maintaining the high catalytic activity of nickel-based phosphides.

### 3.3. Nickel-Based Sulfides

Nickel-based sulfides are abundant in nature and have high electrocatalytic hydrogen evolution activity [17]. Compared with other nickel-based compounds, nickel-based sulfides have the catalytic properties of high conductivity, superior catalytic activity, and high stability. In the nickel-based sulfide crystal structure, the S atom can attract the surrounding electrons of the metal atom due to its high electronegativity, similar to the role of the P atom in nickel-based phosphides, regulating the HER process [51]. The difference between them is that more S content will affect the catalytic activity because the electronegativity of S is greater than that of P. Therefore, researchers have adjusted the electron density in the sulfide lattice by creating S vacancies, thereby indirectly improving its hydrogen evolution performance [52,53]. Additionally, the ratio of S to Ni in nickel-based sulfide also influences its catalytic activity. At present, the common Ni sulfides include NiS, NiS_2_, Ni_3_S_2_, etc., among which the S content in Ni_3_S_2_ is the lowest.

**Figure 2:**
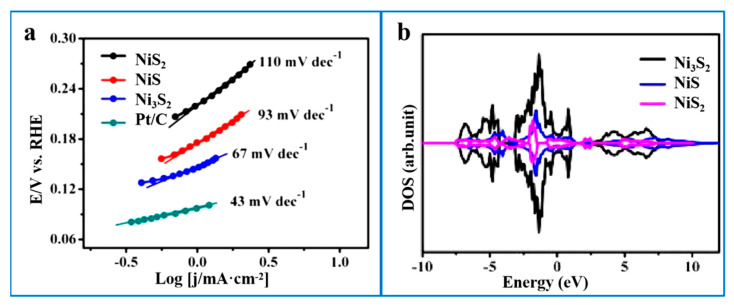
(**a**) HER Tafel plots for NiS_2_, NiS, Ni_3_S_2,_ and Pt/C in 1.0 M KOH. (**b**) The calculated density of states for Ni_3_S_2_, NiS, and NiS_2_ nanocrystals. The Fermi level is defined as 0 eV. Reproduced with permission [54]. Copyright 2019, Royal Society of Chemistry.

Compared with other nickel-based sulfides, Ni_3_S_2_ is a sulfur-deficient nickel sulfide with a higher Ni content, which results in a greater density of exposed metallic active sites on its surface. Its band structure exhibits metallic characteristics with no band gap, endowing it with excellent electrical conductivity to facilitate rapid electron transfer. Moreover, the hexagonal nickelate crystal structure of Ni_3_S_2_ ensures favorable electrochemical stability in both alkaline and neutral media. Ni_3_S_2_ readily forms composite structures with other materials and can be in situ grown on self-supporting conductive substrates, further enhancing its catalytic performance. For example, to compare the electrochemical properties of the three sulfur-nickel compounds, Zheng et al. [54] concluded, by comparing the Tafel slopes of NiS, NiS_2_, and Ni_3_S_2_ compounds at 10 mA cm^−2^, that Ni_3_S_2_ has a Tafel slope of only 67 mV dec^−1^. This is significantly lower than the Tafel slope of NiS and NiS_2_, showing excellent HER performance (Figure 2a). In addition, through DFT calculations, the calculated density of states in Ni_3_S_2_ shows better metal properties, and there is no band gap in its energy band structure, indicating lower resistance and higher conductivity (Figure 2b). This also demonstrates its excellent structural stability and electrical conductivity [55,56]. Therefore, current research on nickel-based sulfides is predominantly focused on Ni_3_S_2_-based catalysts. Table 2 provides a summary of the hydrogen evolution performance of Ni_3_S_2_-based catalysts under different electrolyte conditions. However, during the HER, the chemical adsorption of Ni_3_S_2_ on H* is too strong, which affects the subsequent desorption of H* and the formation of H_2_ [57,58]. Thus, the hydrogen evolution activity of Ni_3_S_2_-based catalysts requires further enhancement and optimization.

## 4. Synthesis Method for Ni_3_S_2_

The initial selection of the preparation method is a critical step to produce an optimal catalyst. It should start by ensuring the controllability and uniformity of the product and guaranteeing the following two aspects: ensuring stable and high-quality chemical composition, and creating more active sites through regulating the morphology and structure. At present, synthetic methods, such as chemical vapor deposition (CVD), electrochemical deposition, and hydrothermal synthesis, are generally used for the preparation of Ni_3_S_2_.

### 4.1. Chemical Vapor Deposition Method

CVD is a method of sublimating and gasifying reactants under high-temperature conditions and at the same time introducing inert gas, protective gas, or reaction gas, and transferring the gasified reactants to the deposition area, where chemical reactions occur and generate a solid product. The Ni_3_S_2_ is synthesized with simple S and Ni or Ni oxides in a tubular atmosphere furnace, generally under high-temperature conditions, where S gasifies and reacts with Ni source compounds to obtain nickel sulfides. Rye et al. [68] placed Ni powder and S powder in a tube furnace with nitrogen gas at a ratio of 3:2, but the obtained particles were easy to agglomerate, resulting in the inability to effectively release active substances during the catalytic process, thereby affecting the catalytic efficiency. Wang et al. [69] placed the flake-like NiO precursor material grown on NF into a tube furnace to vulcanize and synthesize Ni_3_S_2_, which maintained its morphology and improved its conductivity. However, the synthesis of Ni_3_S_2_ materials with special morphologies on a substrate generally requires Ni oxides or hydroxides as precursors, and the synthesis process is relatively complicated, which limits the efficient synthesis and development of electrodes.

### 4.2. Electrochemical Deposition Method

The electrochemical deposition method involves applying an external electric field to drive the transport of cations and anions in the electrolyte, leading to a redox reaction on the electrode that forms a compound. This technique can be used to synthesize Ni_3_S_2_ nanomaterials on a conductive substrate. Wu et al. [70] used nickel foam (NF) as a conductive substrate and modified the morphology and structure of Ni_3_S_2_ nanomaterials by controlling the electrolyte concentration and electrodeposition time to synthesize coral-like Ni_3_S_2_/NF with excellent electrocatalytic performance. Sun et al. [71] synthesized Ni-S compounds with an amorphous structure on NFs with excellent electrochemical performance. However, this method is weakly bound to the substrate, leading to inadequate stability during the catalytic process.

### 4.3. Hydrothermal Reaction Method

The preparation of nanomaterials on substrates by hydrothermal synthesis can obtain materials with uniform distribution, less agglomeration, and ideal stoichiometric composition. Compared with the CVD method, it is milder in synthesis conditions, simpler in the synthesis process, and shows flexibility in adjusting the chemical composition and morphology. Compared with the electrodeposition synthesis method, hydrothermal synthesis can make the active material more tightly combined with the substrate and ensure stability.

Ni_3_S_2_ nanomaterials prepared by a hydrothermal method generally use thiourea or thioacetamide as a sulfur source to react with nickel salt in an aqueous solution. Previous studies have shown that their morphology and structure can be optimized, and their chemical components can be modified by adjusting parameters such as feeding amount, temperature, time, and water content. Cui et al. [72] synthesized Mo-Ni_3_S_2_ catalysts with a rod-like uniform structure on nickel foam by a one-step hydrothermal method. NF acts as both the substrate and a Ni source, while thiourea serves as the sulfur source and reducing agent, reacting with sodium molybdate and nickel foam.

### 4.4. Other Synthesis Methods

Other methods have been applied to prepare Ni_3_S_2_-based electrocatalysts with novel nanostructures, including etching deposition, anion-exchanging strategies, and thermal treatment. A facile method to synthesize efficient catalysts derived from nickel–iron Prussian blue analogs (PBAs) is proposed through the comprehensive utilization of synthetic technologies. Ni(OH)_2_@NiFe PBA/NF nanoarrays are fabricated, firstly, by chemical etching, followed by chemical etching/anion exchange. The mass/charge transport and gas release can be modified by the direct growth of porous nanosheets and three-dimensional (3D) interconnected structures [73]. Thermal treatment typically involves high-temperature annealing of Ni or its precursors (such as Ni(OH)_2_, NiO, or Ni-based precursor films) in a sulfur-rich atmosphere (e.g., sulfur powder, H_2_S gas, or thiourea), facilitating a sulfurization reaction to form Ni_3_S_2_ with good crystallinity. By adjusting the annealing temperature, time, and type of sulfur source, it is possible to optimize the morphology, crystal phase, and electrochemical properties of Ni_3_S_2_. Pola et al. [74] successfully synthesized nickel sulfide dendritic structures by thermally annealing nickel foil, demonstrating the potential of the thermal treatment method for the fabrication of Ni_3_S_2_.

## 5. Strategies for Improving Electrocatalytic Hydrogen Evolution Performance of Ni_3_S_2_-Based Materials

Studies have shown that efficient HER catalysts should have excellent intrinsic activity, active site density, electrical conductivity, and high stability. As a catalyst, Ni_3_S_2_ exhibits high electrical conductivity due to its excellent ratio of Ni atoms to S atoms and unique structure, but the exposed active sites of Ni_3_S_2_ are limited, resulting in unstable electrocatalytic performance. In this paper, strategies to improve the hydrogen evolution performance of Ni_3_S_2_ are studied from four aspects: improving the intrinsic hydrogen evolution activity of the material, increasing the active sites of the catalytic HER, combining Ni_3_S_2_ with other catalysts, and optimizing the electrode structure [75,76].

### 5.1. Improving the Intrinsic Catalytic Activity of Ni_3_S_2_

To enhance the intrinsic activity of Ni_3_S_2_-based electrocatalysts, key strategies include heteroatom doping (e.g., Co, Fe, or P) to optimize the electronic structure and hydrogen adsorption energy (ΔG_H*_), construction of heterostructures (e.g., with MoS_2_ or carbon materials) to take advantage of interfacial synergistic interactions, defect engineering (e.g., sulfur vacancies) to expose the active sites, crystalline facet engineering to preferentially consider highly active surfaces (e.g., (101) facets), and surface functionalization (e.g., phosphide or carbon coatings) to improve stability and charge transfer. Other approaches include the application of strain engineering through substrate interactions, and the use of calculational guides (e.g., DFT) to predict optimal modifications. Together, these approaches aim to tune the electronic environment, increase the active site density, accelerate reaction kinetics, and, often, synergistically combine to improve HER performance. This section will introduce the main methods and their respective advantages and disadvantages.

#### 5.1.1. Doping Heteroatoms

Doping heteroatoms could achieve the following effects: (1) modifying the electronic structure in the Ni_3_S_2_ lattice, tuning the d-band center position, balancing the adsorption and desorption capacity of the catalyst surface for H* during the HER process, and improving the hydrogen evolution efficiency; (2) enhancing of the catalyst’s conductivity; (3) regulating the morphology and structure of the Ni_3_S_2_ catalyst to a certain extent, enriching the active sites of the catalyst [77].

**Figure 3:**
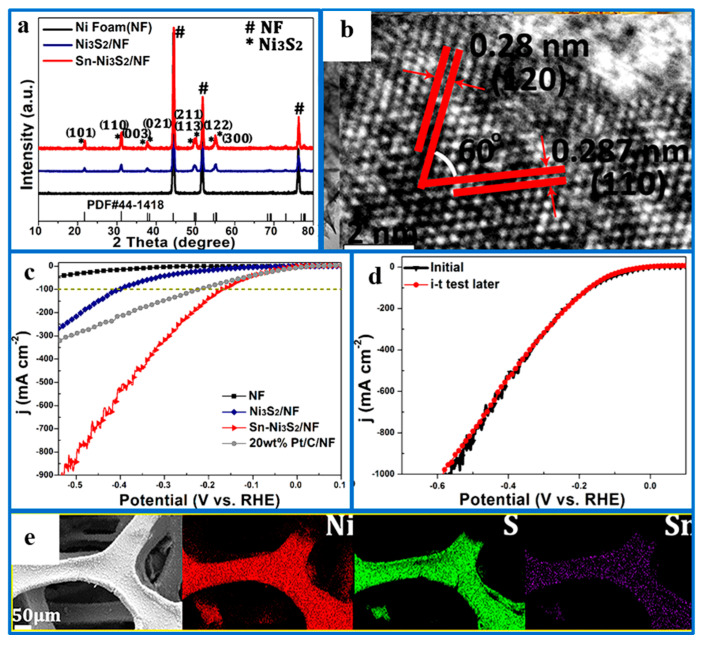
(**a**) XRD patterns of NF, Ni_3_S_2_/NF, and Sn-Ni_3_S_2_/NF (corresponding to Ni_3_S_2_); (**b**) HRTEM graph; (**c**) iR-corrected LSV curves of NF, Ni_3_S_2_/NF, and Sn-Ni_3_S_2_/NF at 20 wt % Pt/C/NF; (**d**) LSV curves of Sn-Ni_3_S_2_/NF before and after the 10,000 CV cycles; (**e**) elemental distribution mapping of Ni, S, and Sn in Sn-Ni_3_S_2_/NF. Reproduced with permission [78]. Copyright 2018, American Chemical Society.

Heteroatom doping can be categorized into metal cation doping and non-metal anion doping. Doping metal cations into the Ni_3_S_2_ lattice can rearrange its electronic structure, creating a configuration that facilitates the adsorption and desorption of H atoms. Yuan et al. [78] synthesized tin (Sn)-doped Ni_3_S_2_/NF with a tremella-like structure as a catalyst active material by hydrothermal method. Experiments showed that Sn doping altered the morphology of the Ni_3_S_2_ catalyst and enhanced its intrinsic activity, resulting in Sn-Ni_3_S_2_/NF exhibiting high catalytic activity and high electrical conductivity for electrochemical activity (Figure 3). The results showed that only 171 mV of overpotential was needed to reach a current density of 100 mA·cm^−2^, with catalytic activity stable for 60 h, indicating that Sn-Ni_3_S_2_/NF displayed excellent electrocatalytic hydrogen evolution performance. This outstanding performance is due to Sn atom incorporation, which regulates the electronic configuration of Ni_3_S_2_, optimizes H* adsorption/desorption on the catalyst surface, enhances catalytic site activity and conductivity, and lowers the energy barrier for electrocatalytic hydrogen evolution.

**Figure 4:**
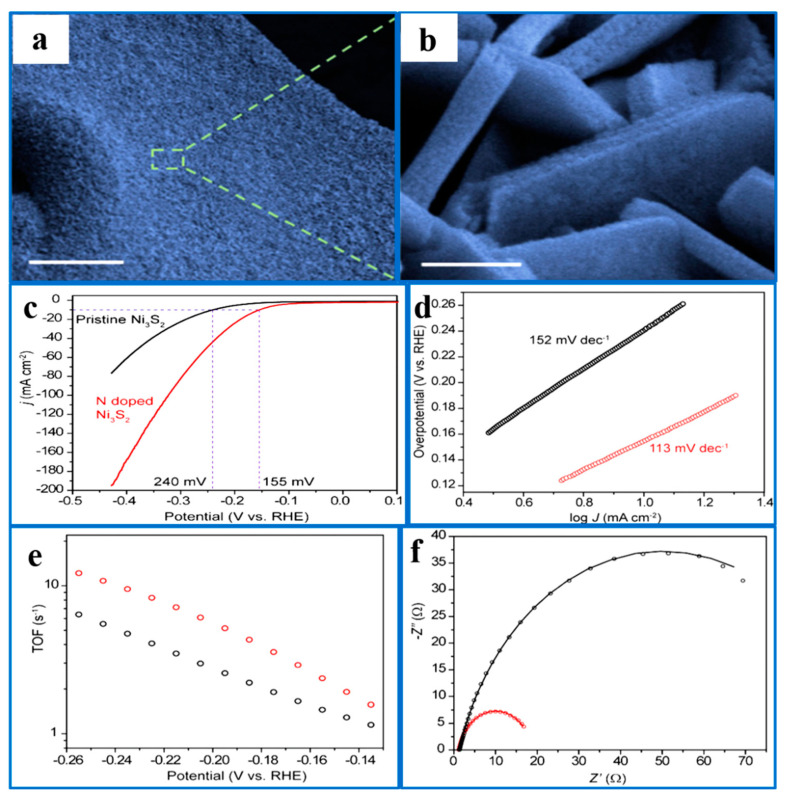
(**a**,**b**) SEM images of the N-doped Ni_3_S_2_ nanosheets on Ni foam, with a scale bar of 20 µm. (**c**) HER polarization curves of the pristine and N-doped Ni_3_S_2_/NF in 1 M KOH at a scan rate of 5 mV·s^−1^. (**d**) Tafel plots of the pristine and N-doped Ni_3_S_2_/NF. (**e**) TOF of the pristine and N-doped Ni_3_S_2_/NF plotted as a function of applied potential. (**f**) Nyquist plot recorded at −0.159 V versus the RHE, with frequencies ranging from 100 kHz to 1 Hz and an amplitude of 5 mV. Reproduced with permission [79]. Copyright 2018, Wiley–VCH.

On the other hand, N-doped into the Ni_3_S_2_ lattice can increase the active sites and change the electronic coordination environment around Ni (Figure 4). Li et al. [79] doped N atoms in Ni_3_S_2_/NF by nitrogen calcination to form N-Ni_3_S_2_/NF, which greatly improved the hydrogen evolution performance. In 1.0 M KOH, the overpotential for a current density of 10 mA·cm^−2^ decreased from 240 mV to 155 mV after nitrogen doping, while the Tafel slope decreased from 152 mV dec^−1^ to 113 mV dec^−1^. DFT calculations revealed that N doping optimizes the ∆G_H*_, increasing active sites and enhancing the hydrogen evolution efficiency of the N-Ni_3_S_2_/NF catalyst.

#### 5.1.2. Introducing Defects

**Figure 5:**
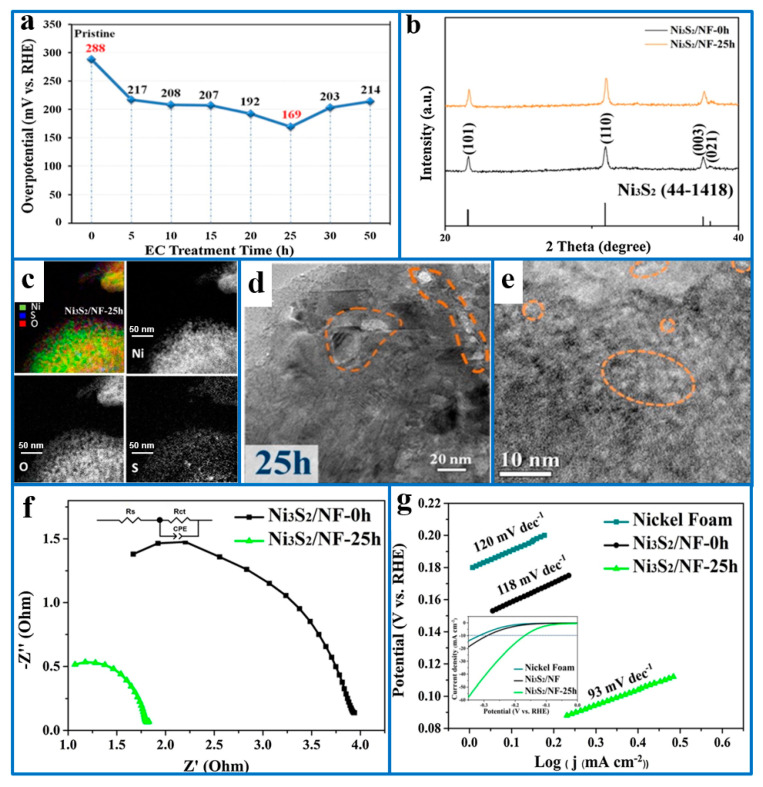
(**a**) The overpotential of each Ni_3_S_2_/NF sample at the respective EC treatment time. (**b**) Enlarged XRD spectra of Ni_3_S_2_/NF-0h and Ni_3_S_2_/NF-25h. Energy-filtered elemental mapping images of (**c**) Ni_3_S_2_/NF-25h. TEM images of (**d**,**e**) Ni_3_S_2_/NF-25h. (**f**) Nyquist plots of Ni_3_S_2_/NF-0h and Ni_3_S_2_/NF-25h. (**g**) Tafel plots of Nickel Foam, Ni_3_S_2_/NF-0h, and Ni_3_S_2_/NF-25h. Reproduced with permission [82]. Copyright 2018, Elsevier.

Studies have shown that the introduction of point defect S vacancies in transition metal sulfides can disrupt the coordination environment of surface atoms in Ni_3_S_2_, thereby enhancing the interaction between the catalyst and reaction intermediates and generating more catalytically active sites. In addition, such defects can modulate the electronic density distribution and improve charge transfer capabilities. At present, most studies on S vacancies are mainly focused on sulfide hydrogen evolution catalysts with layered structures [80,81]. For example, the introduction of sulfur vacancies into MoS_2_ can (1) regulate the electronic structure, reduce the water dissociation barrier, promote the adsorption of H* on the catalyst surface, and promote the formation of HER, and (2) enrich the active sites of the HER. Hao et al. [82] created S vacancies on the surface of Ni_3_S_2_ nanosheets by a simple in situ electrochemical pretreatment, forming an oxygen-doped, sulfur-deficient Ni_3_S_2_ (Ni_3_S_2-x_O_x_) layer. The overpotential of the EC-treated Ni_3_S_2_ electrocatalyst for the HER reduced from 288 mV (pristine Ni_3_S_2_) to 169 mV (EC-Ni_3_S_2_), maintaining its structural and chemical composition for 25 h. This was mainly attributed to the following reasons: (1) the formed Ni_3_S_2-x_O_x_ layer modulated the electronic environment and optimized the adsorption and desorption mechanism of the hydrogen atoms, thereby reducing the overpotential and improving the conductivity, and (2) the poorly crystalline nanosheet or microsphere structure existing in EC-Ni_3_S_2_ increased the abundance of hydrogen-producing active sites on the catalyst surface (Figure 5).

#### 5.1.3. Crystal Facet Engineering

**Figure 6:**
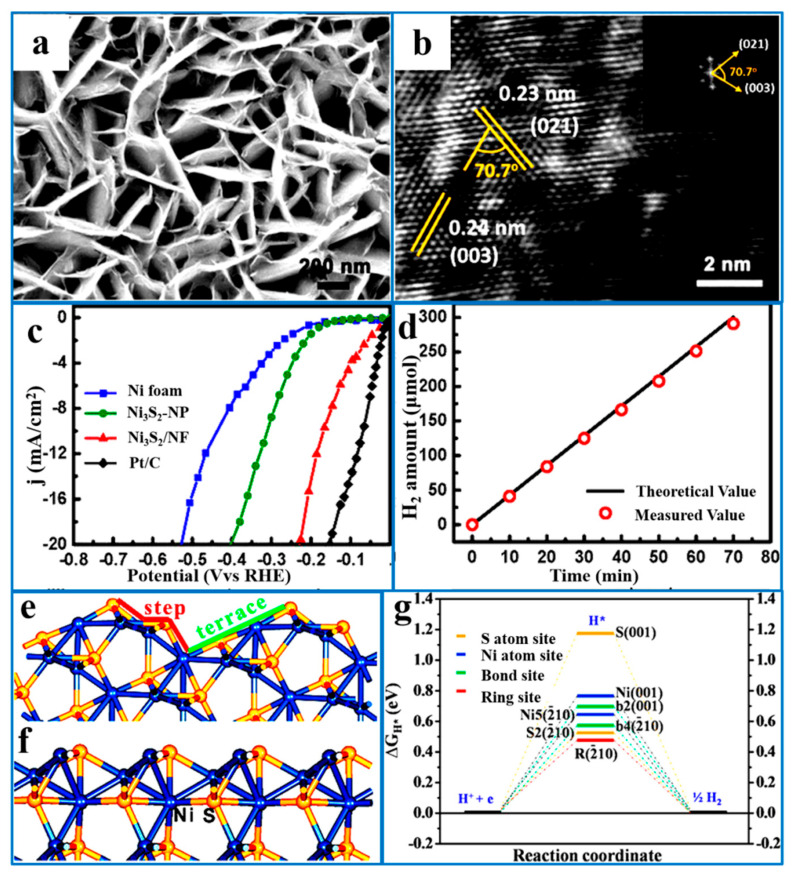
(**a**) SEM images of the Ni_3_S_2_/NF. (**b**) HRTEM image of the Ni_3_S_2_/NF, with the fast Fourier transform image shown in the inset. (**c**) The steady-state current density vs. the applied voltage during the HER at pH7 for NF, Ni_3_S_2_ nanoparticles, Ni_3_S_2_/NF, and Pt/C (20 wt%). (**d**) The electrocatalytic efficiency of H_2_ production over the Ni_3_S_2_/NF at a current density of ca. 20 mA·cm^−2^, measured for 70 min. The most stable terminations of$\;\left\{\bar{2}10\right\}$ (**e**) and $\left\{001\right\}$ (**f**) surfaces of Ni_3_S_2_. (**g**) The calculated free-energy diagram of the HER over $\left\{\bar{2}10\right\}$ and $\left\{001\right\}$ surfaces at the equilibrium potential. The Ni and S atoms of Ni_3_S_2_ are represented by the blue and yellow spheres, respectively. Reproduced with permission [83]. Copyright 2015, American Chemical Society.

According to the crystal structure characteristics of Ni_3_S_2_, a catalytically active crystal facet can be rationally designed and selectively exposed to enhance the hydrogen evolution activity of the catalyst. This facet engineering strategy not only increases the number of active sites but also optimizes the catalytic reaction pathway, thereby improving both the reaction kinetics and overall electrocatalytic efficiency. Zou et al. [83] synthesized high-index nanosheet arrays on NF. The results showed that Ni_3_S_2_/NF required 223 mV of overpotential to achieve a current density of 10 mA·cm^−2^ due to enriched S and Ni active sites on high-index crystal surfaces, indicating its stable and efficient electrocatalytic activity. Therefore, the method could provide higher catalytic activity compared with that of low-index crystal planes. In addition, the synergistic catalytic effect of the synthesized high-index nanoarrays also enhances the catalytic activity (Figure 6).

Tu et al. [84] compounded Ni_3_S_2_ with TiO_2_ with exposed crystal facets using a low-temperature vulcanization method and synthesized a Ni_3_S_2_@TiO_2_ sheet structure hydrogen evolution catalyst on NF without a binder. In this work, various optimization strategies were applied to enhance the hydrogen evolution activity of the catalyst, achieving an overpotential of just 112 mV. This was due to the effective exposure of the high hydrogen evolution activity crystal facets of Ni_3_S_2_ and the synergistic effect of the Ni_3_S_2_ and TiO_2_ phase recombination to improve the catalytic activity. Meanwhile, the catalyst had a uniform and porous nanosheet structure, which provided a larger specific surface area for active substances.

#### 5.1.4. Synergistic Effects

The intrinsic activity of Ni_3_S_2_ catalysts can be effectively enhanced through three primary strategies: heteroatom doping, defect engineering, and crystal facet engineering. These approaches not only exhibit individual effectiveness but also demonstrate significant synergistic effects by jointly modulating the structural and electronic properties of the catalyst, thereby achieving comprehensive performance optimization.

Heteroatom doping often disrupts the local symmetry of the Ni_3_S_2_ lattice, leading to the formation of structural defects, such as S vacancies and metal vacancies. These defects act as new active sites and further induce electronic inhomogeneity, which helps to adjust the ∆G_H*_ towards more favorable values. Additionally, the dopant atoms can increase the local electronic density in defect-rich regions or generate heterostructured electronic configurations, thereby facilitating the formation of efficient electron coupling channels. This enhances localized electron mobility at the catalytic interface and accelerates the kinetics of the HER [85].

Moreover, dopant atoms tend to preferentially occupy high-energy or specific crystallographic facets, altering the surface atomic arrangement and the density of states. The charge redistribution induced by doping also enhances the adsorption capability of these facets towards reactant molecules, further amplifying the effectiveness of facet engineering. High-index facets are naturally enriched with unsaturated coordination sites and edge structures. When defect engineering is applied to these regions—such as by introducing S vacancies—higher densities of active sites can be achieved. These defect-enriched zones, guided by facet engineering, provide optimized platforms with tailored electronic structures for catalytic reactions, thus significantly increasing the density of active sites per unit area and improving overall HER kinetics [16,83,86,87,88,89]. Therefore, multi-dimensional synergistic modulation strategies hold great potential for substantially enhancing the intrinsic activity of Ni_3_S_2_-based electrocatalysts.

### 5.2. Increase the Number of Catalytic Active Sites in Ni_3_S_2_-Based Materials

To improve the hydrogen evolution activity of Ni_3_S_2_-based catalysts, good physical properties should also be constructed to expose more active sites. To this end, the following strategies are feasible: (1) controlling morphology, such as building thinner lamellar structures and constructing porous or hollow structures, and (2) synthesizing amorphous Ni_3_S_2_ or reducing the crystallinity of materials.

**Figure 7:**
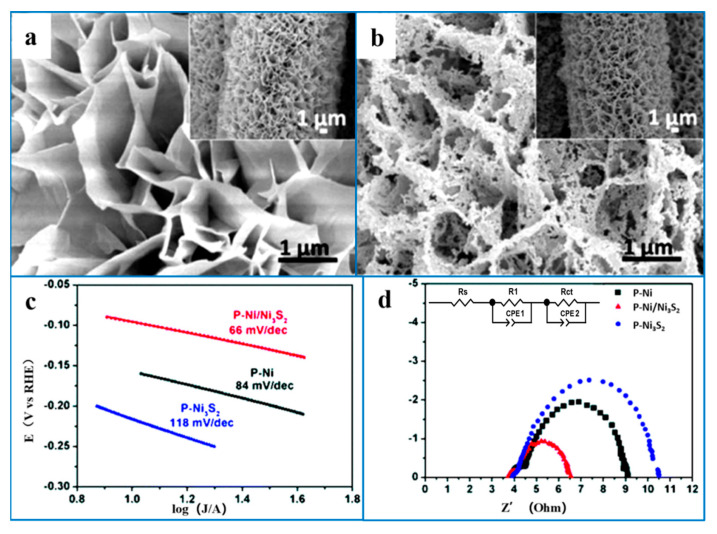
SEM images of (**a**) the Ni(OH)_x_S_y_ nanosheet precursor and (**b**) P-Ni/Ni_3_S_2_ derived from the reduction of Ni(OH)_x_S_y_ nanosheets. (**c**) Tafel plots, and (**d**) Nyquist plots of P-Ni, P-Ni_3_S_2_, and P-Ni/Ni_3_S_2_ on OCC at an overpotential of 170 mV. Reproduced with permission [90]. Copyright 2017, Royal Society of Chemistry.

The morphology and structure can affect the specific surface area and the number of active sites in nano-Ni_3_S_2_ materials. For example, Ni_3_S_2_ catalysts with porous structures, ultra-thin nanosheet structures, hollow structures, and other morphology structures can increase the ECSA of electrocatalysts, which is conducive to the diffusion and transport of substances. In addition, good morphology and structure can facilitate the escape of H_2_ and improve the efficiency of hydrogen evolution. For example, Wu et al. [84] successfully synthesized Ni_3_S_2_/NF with a coral-like structure by electrodeposition, improving the electrocatalytic performance by controlling the morphology. Yang et al. [90] developed a three-dimensional porous Ni/Ni_3_S_2_ nano-network on nickel foam as an electrocatalyst. In an alkaline medium, an overpotential of 95 mV was needed to reach a current density of 10 mA·cm^−2^, demonstrating exceptional HER activity. This enhanced performance can be attributed to the formation of a nano-network structure, which increases the number of active sites, facilitates electrolyte contact and material transport, and induces a synergistic effect between Ni atoms and Ni_3_S_2_, thereby significantly boosting catalytic activity (Figure 7).

On the other hand, amorphous or weakly crystalline catalysts, with numerous unsaturated chemical bonds and defect sites, expose more active sites, enhancing the adsorption of HER intermediates at the electrode/electrolyte interface [91,92]. Li et al. [43] were able to control the formation of amorphous Ni_3_S_2_ by Mo doping using solid-state fusion synthesis. The results showed that doping Mo atoms could trigger the formation of amorphous Ni_3_S_2_ nanosheets and could also regulate the electronic configuration of Ni_3_S_2_ and enhance the intrinsic activity of hydrogen evolution. The results showed that the degree of the amorphous state of Ni_3_S_2_ could be tuned by adjusting the feeding amount of the Mo source. With an appropriate amount of Mo, Mo-Ni_3_S_2_ could form an amorphous state, which possessed the strongest adsorption capacity for the intermediate medium of the HER, leading to excellent HER performance.

### 5.3. Combining Ni_3_S_2_ with Other Catalysts

In addition, combining Ni_3_S_2_ with other catalytically active materials to form a multi-component catalytic could create a synergistic effect, significantly reducing the reaction kinetic barrier and improving the HER and its efficiency [93,94]. This section mainly discusses the combination of Ni_3_S_2_ with transition metals, metal sulfides, and other catalysts to improve the hydrogen evolution performance.

**Figure 8:**
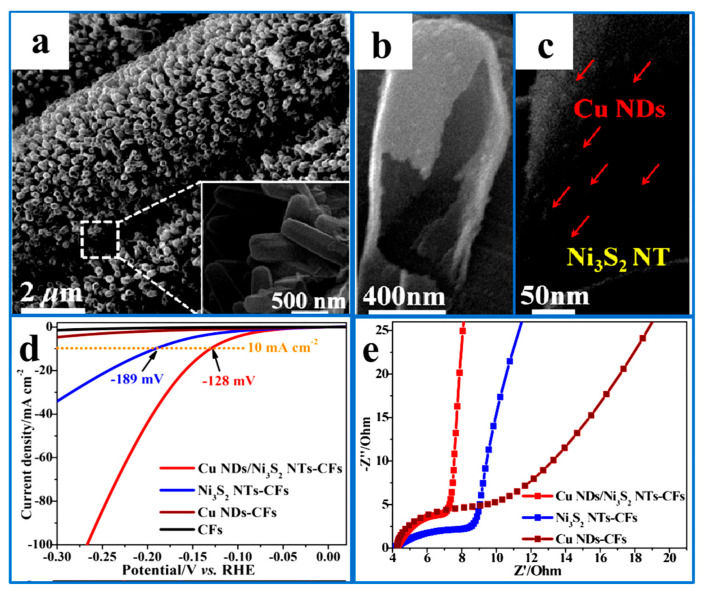
(**a**–**c**) SEM images of Cu NDs/oNi_3_S_2_ NTs-CFs. (**d**) Polarization curves. (**e**) Nyquist plots. Reproduced with permission [95]. Copyright 2018, American Chemical Society.

Li et al. [95] combined Ni_3_S_2_ with transition metal Cu nanodots to obtain a Cu-Ni_3_S_2_ catalyst with a low onset potential, high stability, and excellent electrical conductivity. The Cu-Ni_3_S_2_ catalyst required an initial overpotential of 60 mV to reach 10 mA·cm^−2^, showing excellent hydrogen evolution performance because its hierarchical structure was conducive to mass diffusion in the HER process. In addition, the addition of Cu facilitated the regulation of the adsorption of S on the surface of the catalyst. Meanwhile, Cu nanodots can induce electronic interactions with Ni_3_S_2_, rendering Ni_3_S_2_ negatively charged, and Cu positively charged. This charge redistribution improves the chemical adsorption of H_2_O molecules on the Cu-Ni_3_S_2_ catalyst, optimizes the adsorption and desorption of H* intermediates, and facilitates the Volmer and Heyrovsky steps, thereby enhancing the overall HER activity (Figure 8).

**Figure 9:**
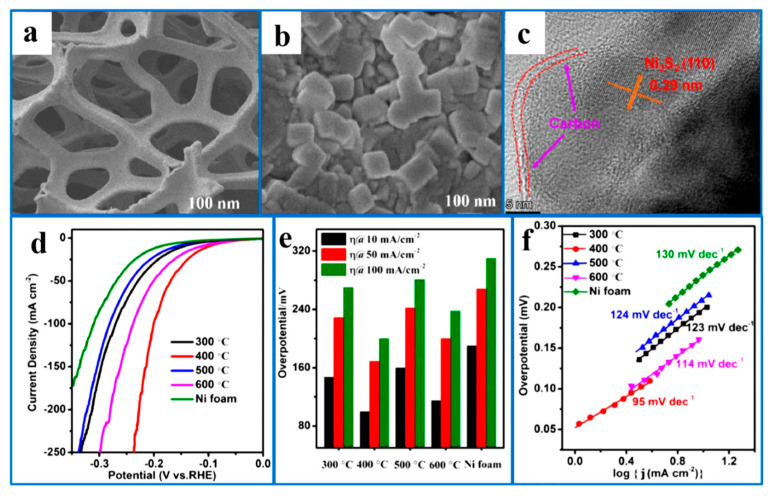
(**a**,**b**) SEM images and (**c**) TEM image of N-Ni_3_S_2_ @NG/NF (400 °C); (**d**) polarization curves (iR-corrected) at a scan rate of 5 mV·s^−1^; (**e**) overpotential plot; and (**f**) Tafel plots of different samples, respectively. Reproduced with permission [96]. Copyright 2021, Elsevier.

In addition, Ni_3_S_2_ could also be combined with non-metals to improve hydrogen evolution performance. Li et al. [96] used CVD technology to dope non-metallic N into Ni_3_S_2_ crystals and synthesized N-doped graphene-coated Ni_3_S_2_ nanocubes (N-Ni_3_S_2_@NG). N-Ni_3_S_2_@NG required an overpotential of only 100 mV at a current density of 10 mA·cm^−2^, with a working voltage of 1.53 V, indicating its exceptional performance. Moreover, its ultra-stable catalytic activity was achieved by a significant number of active center electrons and proton transfer channels provided by the nano-cubic structure, thereby improving its electron transport performance (Figure 9).

**Figure 10:**
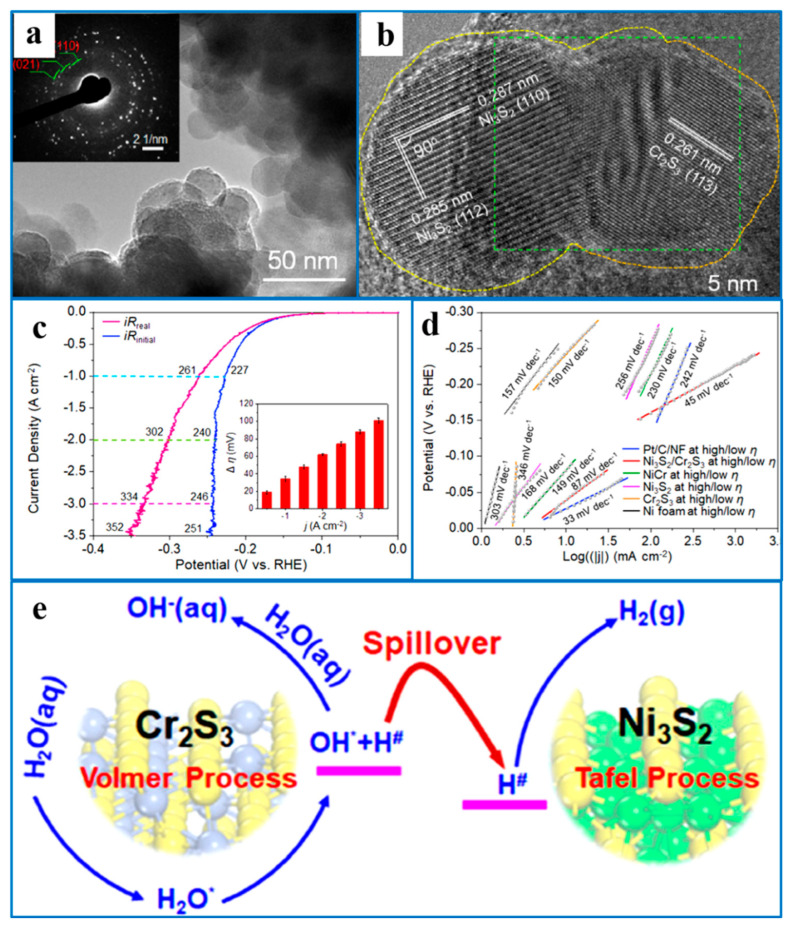
(**a**) TEM image and the corresponding SAED pattern (inset), (**b**) HRTEM image of Ni_3_S_2_/Cr_2_S_3_@NF, (**c**) the LSV curves of Ni_3_S_2_/Cr_2_S_3_@NF compensated, respectively, by iRinitial (pink) and iRreal (blue), with an inset showing the overpotential differences between the iRinitial- and iRreal-compensated LSV curves under the corresponding current densities, (**d**) Tafel plots of Ni_3_S_2_/Cr_2_S_3_@NF, NiCr@NF, Ni_3_S_2_@NF, Cr_2_S_3_@NF, and PtC@NF, and (**e**) a schematic representation of the proposed reaction mechanism for Ni_3_S_2_/Cr_2_S_3_@NF. Reproduced with permission [97]. Copyright 2022, American Chemical Society.

Combining with other transition metal sulfides, the construction of new compounds, such as Ni_3_S_2_, could also be used to improve HER activity. Fu et al. [97] used electrodeposition–sulfurization synthesis to grow synergistically hybridized Ni_3_S_2_/Cr_2_S_3_ nanoparticles on NF (Figure 10a,b). The catalyst could resist the inhibition caused by high-concentration hydrogen coverage in electrolytic water splitting, improving the transfer rate of H* from hydrogen-rich sites to hydrogen-deficient sites, thereby improving the efficiency of electrocatalytic reactions. Figure 10c shows that the co-existence of the two components, Ni_3_S_2_ and Cr_2_S_3_, leads to good HER electrocatalytic activity, and Figure 10d shows that Ni_3_S_2_/Cr_2_S_3_@NF has excellent HER compared to Pt/C@NF under high current density environment kinetic properties, as shown by its low overpotential. Figure 10e illustrates the possible reaction pathway of the alkaline HER occurring on the catalyst surface. Initially, the active sites on the Cr_2_S_3_ component strongly adsorb H_2_O molecules, facilitating their dissociation into H*. Subsequently, the dissociated H* migrates across the interface and combines with H* on the Ni_3_S_2_ surface to form H_2_.

**Figure 11:**
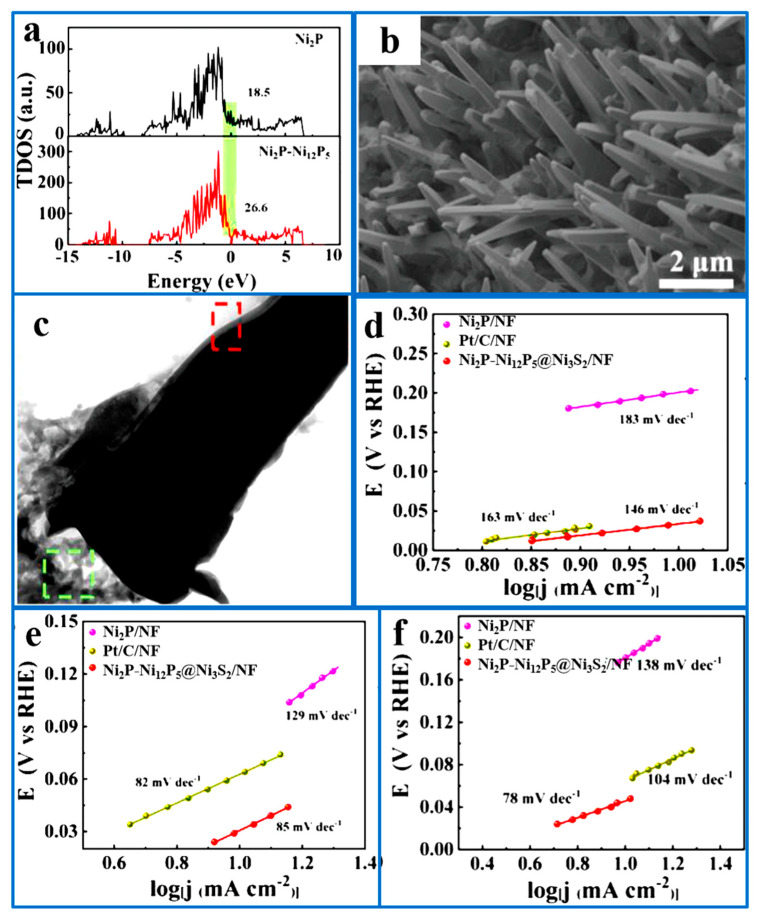
(**a**) The calculated total DOS (TDOS) of these two TMPs; (**b**,**c**) FESEM images of sequential phase-converted Ni_2_P-Ni_12_P_5_@Ni_3_S_2_/NF and corresponding Tafel plots in (**d**) 1 M PBS, (**e**) 1 M KOH, and (**f**) 0.5 M H_2_SO_4_. Reproduced with permission [98]. Copyright 2022, Wiley–VCH.

Yang et al. [98] established a hierarchical structure of Ni_2_P-Ni_12_P_5_ nanorod arrays on Ni_3_S_2_ film substrates by sulfidation and corrosion, followed by phosphating nickel foam, to synthesize Ni_2_P-Ni_12_P_5_@Ni_3_S_2_/NF as a catalyst for water electrolysis. It was found that Ni_2_P-Ni_12_P_5_ was vertically arranged, presenting a nanorod array, and the inner diameter gradually decreased from the root to the end (Figure 11b). Ni_2_P-Ni_12_P_5_@Ni_3_S_2_/NF showed similar morphology, as evidenced by Figure 11c, and therefore has the same advantages. This spatial complexity of the structure created a larger specific surface area, meaning that more active sites were available for better ion transportability. In addition, compared with Pt/C/NF, Ni_2_P/NF, and Ni_3_S_2_/NF, Ni_2_P-Ni_12_P_5_@Ni_3_S_2_/NF had the best HER catalytic performance, as evidenced by the smallest Tafel slope (Figure 11d), owing to the interfacial coupling induced synergy between Ni_12_P_5_ and Ni_2_P. Moreover, it was found that Ni_2_P-Ni_12_P_5_@Ni_3_S_2_/NF exhibited excellent electrocatalytic performance under both acidic and alkaline conditions, as compared with TMP electrocatalysts in other studies, which had great significance in expanding the pH application range of HER electrocatalysts (Figure 11).

### 5.4. Loading Ni_3_S_2_ on a Self-Supporting Conductive Substrate

In the research on the regulation and control of the hydrogen evolution activity of Ni_3_S_2_ nanostructure catalysts, there is still a very important link to how to effectively assemble them into catalytic electrodes for practical applications [43]. Studies have demonstrated that when Ni_3_S_2_ catalytic materials are directly synthesized on a self-supporting conductive substrate, the resulting Ni_3_S_2_ nanostructure could be effectively anchored to the support, preventing its accumulation and aggregation. This stabilization enhances the exposure of active sites and promotes the migration of active species and electrons during the catalytic process. Consequently, these factors collectively contribute to an improvement in catalytic activity [91,92,93].

**Figure 12:**
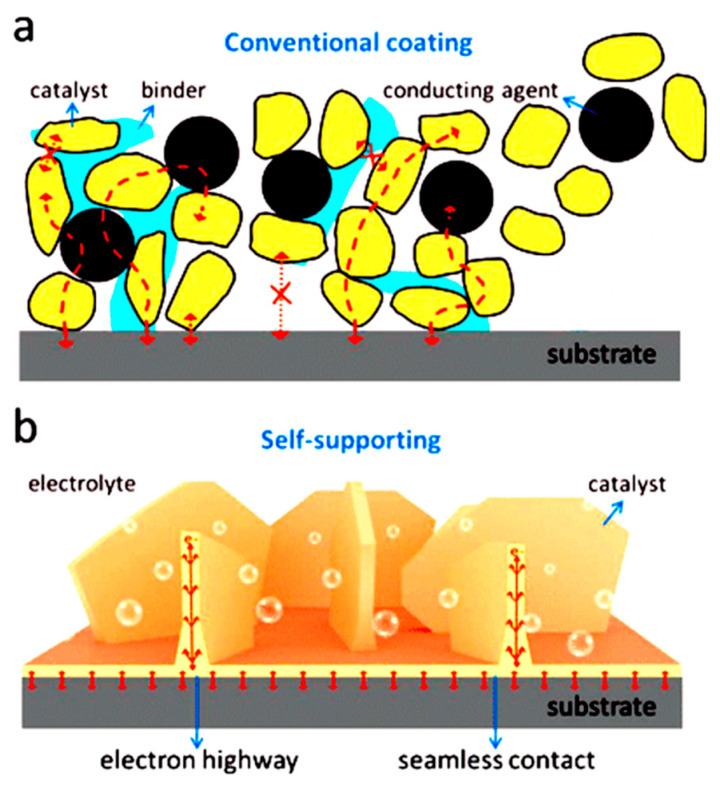
The difference between the traditional powder-like assembled electrodes and the electrode structures using self-supporting conductive substrates in the catalytic process: (**a**) electrodes assembled with traditional powder-like catalysts and (**b**) catalysts grown directly on self-supporting substrates. Reproduced with permission [99]. Copyright 2020, Wiley–VCH.

Figure 12 shows the difference between the traditional powder-like assembly electrode and the electrode structure using a self-supporting conductive substrate in the catalytic process [99]. Traditional powdered catalysts require the use of binders during electrocatalytic testing and application, however, binders can block the contact between active materials and hinder the diffusion of active materials during catalysis. Therefore, the electron and material transport of the electrode during the catalytic process can be effectively enhanced by directly loading the catalyst nanomaterials on the self-supporting conductive substrate, eliminating the use of binders, and thereby improving the efficiency of the hydrogen evolution reaction. Moreover, the catalyst material has strong adhesion to the substrate, which is not prone to peeling and separation and can provide long-term catalytic stability. The currently used self-supporting conductive substrates mainly include carbon materials, such as graphene or carbon nanotubes, metal materials, such as Ni, Cu, or Fe, and other forms of conductive materials, such as fluorine-doped tin oxide.

**Figure 13:**
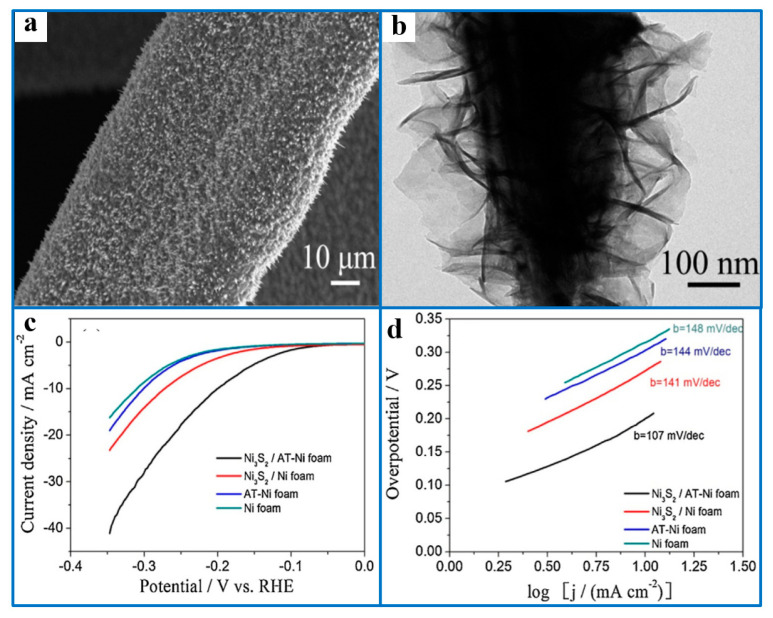
(**a**) Low-magnification SEM images of Ni_3_S_2_/AT-Ni foam. (**b**) Low-magnification TEM images of Ni_3_S_2_/AT-Ni foam. HER performance of as-synthesized samples. (**c**) LSV for Ni_3_S_2_/AT-Ni foam, Ni_3_S_2_/Ni foam, AT-Ni foam, and Ni foam in 1 M KOH solution at a scan rate of 2 mV·s^−1^. (**d**) Corresponding Tafel plots of Ni_3_S_2_/AT-Ni foam, Ni_3_S_2_/Ni foam, AT-Ni foam, and Ni foam. Reproduced with permission [103]. Copyright 2015, Elsevier.

As a transition metal, the Ni substrate not only plays a supporting role but also facilitates charge transport in catalytic reactions due to its good electrical conductivity. NF has a porous structure with a 3D framework and a large electrode surface area, which can provide a large contact area for the catalyst and electrolyte. In recent years, the direct fabrication of catalytic materials on metal substrates (such as NF) with three-dimensional (3D) structural frameworks as electrocatalytic reaction devices has been extensively studied [95,96,97,98,100,101,102]. Wang et al. [103] synthesized rod-shaped porous Ni_3_S_2_ nanomaterials on acid-treated nickel foam using a hydrothermal method. The effective charge transport in the NF framework greatly enhanced the electrocatalytic reaction activity of the Ni_3_S_2_ powder (Figure 13). Specifically, the overpotential required for the HER decreased from 300 mV to 200 mV, and the catalytic activity remained almost unchanged after a long-term stability test. The excellent catalytic activity and stability can be attributed to the fact that the rod-shaped porous Ni_3_S_2_ catalyst supported on NF can provide a large electrochemically active specific surface area, and the good binding force between Ni_3_S_2_ and NF can maintain long-term catalytic activity.

## 6. Conclusions, Challenges, and Perspectives

Hydrogen energy, a clean source with high efficiency, has gained significant attention, particularly hydrogen production through water electrolysis, due to its low pollution. However, noble metal catalysts used in this process are costly. To enable large-scale production, there is a need to develop low-cost, high-efficiency electrocatalysts. Ni-based catalysts are promising due to their good hydrogen evolution performance. Among them, compared with other Ni-based catalysts, Ni_3_S_2_ exhibits better hydrogen evolution activity due to its unique chemical structure. However, its high overpotential makes it difficult to put it into industrial production. Therefore, this paper summarizes improvements in the synthesis, microscopic mechanism, and electrocatalytic hydrogen evolution performance of Ni_3_S_2_ composites. The main strategies for modifying composite Ni_3_S_2_ are also highlighted. These strategies focus on four key aspects: enhancing intrinsic catalytic activity, increasing the number of active sites, constructing heterostructures, and loading Ni_3_S_2_ on self-supporting conductive substrates. Through these strategies, the morphology and structure of Ni_3_S_2_-based materials are deliberately modified to increase their specific surface area and active sites, and the synergy between atoms is intentionally constructed. As a result, the overpotential required for the modified Ni_3_S_2_-based material to achieve 10 mA·cm^−2^ in the water electrolysis reaction is significantly reduced, the Tafel slope decreases, and stability is improved, demonstrating exceptional catalytic activity. Up to now, challenges still exist that hinder the application of Ni_3_S_2_-based catalysts. These are summarized as follows, combined with prospects.

1. The innovation of synthesis strategies will be crucial for enhancing the performance of Ni_3_S_2_ catalysts, particularly through advanced methods, such as in situ assembly and atomic layer deposition (ALD), which allow for precise control over the catalyst’s structure and composition. In addition, the development of synthesis methods suitable for large-scale industrial production remains to be further optimized. Among them, the electrodeposition method could be a promising approach due to its easily controlled operational conditions. However, the current lack of comprehensive technological procedures and standards limits its practical implementation, which warrants focused attention in future research.
2. Additionally, the catalytic mechanism of Ni_3_S_2_-based catalysts remains a topic of ongoing debate, and identifying the true active centers or phases is of great significance for their further development. Future research should prioritize in-depth investigations using computational methods, such as DFT, to gain valuable insights into the catalytic mechanism, predict potential high-efficiency active sites, and provide theoretical guidance for experimental design, thereby accelerating the development and application of high-performance Ni_3_S_2_ catalysts. In addition, the advancement of in situ characterization techniques is essential for monitoring the dynamic evolution of active centers during electrochemical reactions, which will contribute to a deeper understanding of structure–activity relationships.
3. The electrocatalytic activity and stability of Ni_3_S_2_-based catalysts still fall short of meeting industrial requirements, particularly under conditions of high current density, elevated temperatures, and strongly alkaline environments. Therefore, future research will focus on designing Ni_3_S_2_-based catalysts with excellent anti-corrosion and anti-degradation properties to enhance their durability and longevity in practical applications.

## Figures and Tables

**Table 1:** Advantages and disadvantages of various nickel-based compounds as electrocatalysts.

Electrocatalyst	Advantage	Disadvantage	References
Nickel-based sulfide	Rich reserves; High conductivity; High stability.	Unbalanced hydrogen evolution performance.	[38]
Nickel-based oxide	Low toxicity; Low cost; Rich reserves.	Poor conductivity; Poor hydrogen evolution activity.	[39]
Nickel-based phosphide	Easy to synthesize; High conductivity.	Low stability; Slightly toxic.	[40]
Nickel-based nitride	Strong corrosion resistance; The structure is easy to control.	Difficult to synthesize.	[38,41]
Nickel-based carbide	Strong electrical conductivity; Good stability.	Polluted environment.	[42]

**Table 2:** Comparison of the catalytic performance in the Ni_3_S_2_-based electrocatalysts.

Catalyst	Electrolyte	Overpotential [mV]@Current Density [mA cm^−2^]	Stability Test	References
N-Ni_3_S_2_/NF	1 M KOH	110@10	10 h at ~50 mA cm^−2^	[59]
W-doped Ni_3_S_2_-NiFeLaOH	1 M KOH	67@10	40 h at 10 mA cm^−2^	[60]
Co-Ni_3_S_2_/NF	0.5 M H_2_SO_4_	206@100	11 h at 100 mA cm^−2^	[61]
Ni_3_S_2_/Ni foam	1 M PBS 1 M KOH	220@10, 396@100 123@10, 260@100	10 h at ~15mA cm^−2^ 10 h at ~68 mA cm^−2^	[62]
Ni_3_S_2_-WO_3_/NF-1	0.5 M H_2_SO_4_ 1 M KOH	86@10 107@10	12 h at 10 mA cm^−2^ 12 h at 10 mA cm^−2^	[63]
Ni_3_S_2_@NPC	0.5 M H_2_SO_4_ 1 M KOH 2 M PBS	91.6@10 60.8@10 193.0@2	111 h at ~50 mA cm^−2^ 28 h at ~50 mA cm^−2^ 28 h at ~50 mA cm^−2^	[64]
NiCo_2_O_4_/Ni_3_S_2_/NF	0.5 M H_2_SO_4_ 1 M KOH	184@10 140@10	14 h at 10 mA cm^−2^ 14 h at 10 mA cm^−2^	[65]
Ni_3_S_2_/NC20	0.5 M H_2_SO_4_ 1 M KOH	174@10 199@10	15 h at 10 mA cm^−2^ 15 h at 10 mA cm^−2^	[66]
1D N-Ni_3_S_2_	0.5 M H_2_SO_4_ 1 M KOH	196@10 105@10	12 h at 10 mA cm^−2^ 12 h at 10 mA cm^−2^	[67]
